# Heritability of objectively assessed and self‐reported sedentary behavior

**DOI:** 10.1111/sms.13658

**Published:** 2020-04-06

**Authors:** Nienke M. Schutte, Charlotte Huppertz, Stieneke Doornweerd, Meike Bartels, Eco J.C. de Geus, Hidde P. van der Ploeg

**Affiliations:** ^1^ Department of Biological Psychology Netherlands Twin Register Vrije Universiteit Amsterdam Amsterdam The Netherlands; ^2^ Amsterdam Public Health Research Institute Amsterdam University Medical Centres Amsterdam The Netherlands; ^3^ Department of Psychiatry, Psychotherapy and Psychosomatics Medical Faculty RWTH Aachen Aachen Germany; ^4^ Department of Internal Medicine Amsterdam UMC Location VU University Medical Center Amsterdam The Netherlands; ^5^ Department of Public & Occupational Health Amsterdam UMC Location VU University Medical Center Amsterdam The Netherlands

**Keywords:** bivariate, genetics, MVPA, sitting, twin study

## Abstract

Understanding the sources of the large individual differences in sedentary behavior is of great importance as this behavior is associated with pre‐mature mortality and non‐communicable diseases. Here, we report on the contribution of genetic and environmental factors to the variation in objectively assessed (accelerometer) sedentary behavior and self‐reported sitting and their shared genetic basis. In addition, the overlap of the genetic risk factors influencing sedentary time and moderate‐to‐vigorous physical activity (MVPA) was estimated. A sample of 800 individuals (twins and their siblings) was equipped with an Actigraph accelerometer for 7 days and reported on their sitting time and time spent on MVPA on those days using the IPAQ‐SF. Genetic factors explained 56% (CI: 44%, 65%) of the individual differences in objective sedentary behavior (Actigraph) and 26% (CI: 0%, 51%) of the individual differences in self‐reported sedentary behavior (IPAQ‐SF). A modest correlation (0.33) was found between these measures, which was for 45% accounted for by genetic influences. The genetic correlation was 0.49 reflecting a partly overlapping set of genes that influenced both measurements. A modest correlation (−0.27) between Actigraph‐derived sedentary time and MVPA was found, which was 13% accounted for by genetic effects. The genetic correlation was −0.31, indicating that there are overlapping genetic variants that increase sedentary time and decrease MVPA or vice versa. To conclude, more than half of the individual differences in objective sedentary time could be attributed to genetic differences, while for self‐reported sitting this was much lower. In addition, using objective measurements, this study confirms that sedentary time is not simply the inverse of MVPA. Future studies are needed to understand the pathways translating genomic variation into variation in these behaviors and how this knowledge might feed into the development of health promotion interventions.

## 1 INTRODUCTION

Sedentary behavior is defined as any waking behavior characterized by an energy expenditure ≤1.5 metabolic equivalents (METs), while in a sitting, reclining, or lying posture.^1^ Sedentary behavior can be measured subjectively with self‐report questionnaires as well as objectively with activity monitors.^2^ Systemic reviews of studies that aimed to quantify the link between daily sitting and adverse health outcomes show that sedentary behavior is associated with pre‐mature mortality and the development of a variety of non‐communicable diseases, including type 2 diabetes and cardiovascular diseases.^3^, ^4^, ^5^ Shedding light on the sources of the individual differences in sedentary behavior might aid our understanding of the etiology of this trait.^6^ Furthermore, by identifying the biological pathways and determinants leading to sedentary behavior, we can better map targets for interventions intended to reduce and/or interrupt the time spent in sedentary behaviors.^7^, ^8^

Many environmental influences, including transportation and work‐related demands, may impact on sedentary behavior. However, differences in intrinsic, biological factors in the regulation of levels of sedentary behaviors might also play an important role. Studies in nuclear families and twins provided evidence that part of these individual differences result from differences in genetic make‐up.^9^, ^10^ Familial aggregation of a sedentary lifestyle can be investigated by computing correlations among relatives, such as siblings, parents, and their offspring, from which the relative importance of genetic and environmental factors to the observed individual differences in sedentary behavior can be estimated. An even more powerful design to disentangle the relative importance of environmental and genetic influences on a trait or behavior is the twin design. This design includes both genetically identical, or monozygotic (MZ), twins and non‐identical, or dizygotic (DZ), twins and allows for separating the genetic influences (referred to as “A” for additive genetic influences and “D” for dominant genetic influences), shared environmental influences (influences shared with other family members eg upbringing; referred to as “common” or ‘C’) and unique environmental influences (influences that are specific to the individual; referred to as “E”).

Only a few studies employed a twin design to estimate the heritability of sedentary behavior. Kujala et al. (2002) reported a heritability of 50% for doing sedentary work in an adult Finnish cohort aged 24‐60 years.^9^, ^10^ In an older Finnish twin cohort (aged 53‐67 years), a heritability of 35% was found for total self‐reported sitting time^11^ and more recently a heritability of 41% was reported for objectively measured sedentary behavior in older Finnish twins (aged 71‐75 years).^12^ In a sample of Dutch adolescent twins and their siblings, it was reported that variation in sedentary behavior among 12‐year‐olds was accounted for by genetic (boys: 35%; girls: 19%), shared environmental (boys: 29%; girls: 48%), and nonshared environmental (boys: 36%; girls: 34%) factors. Variation in sedentary behavior among 20‐year‐olds was accounted for by genetic (boys: 48%; girls: 34%) and nonshared environmental (boys: 52%; girls: 66%) factors.^13^ All of these studies reported the heritability of self‐reported sedentary work or sitting time. As questionnaires are relatively easier and cheaper to use in large scale epidemiological studies than objective measurement tools, studies on the heritability of *objectively* measured sedentary time are scarce. A twin study in children aged 9‐12 years showed no genetic contribution to *objectively* measured sedentary using accelerometry.^14^ One study objectively measured sedentary time in a large sample of 770 adult twin pairs using combined heart rate recording and a uniaxial accelerometer on the trunk (Actiheart) and reported a heritability estimate of 47% in a sample with a mean age of 56.^15^

Based on the scarcity of studies with objectively assessed sedentary behavior, it is unclear whether the genetic and environmental factors that influence self‐reported sedentary behavior correspond to those that influence objectively measured sitting time. For instance, heritable personality traits may directly influence sedentary behavior and this would be detected by either subjective or objective assessment. If personality also leads to bias in self‐report, this would indirectly influence subjective, but not objective, measurement, leading to an imperfect genetic correlation between subjective and objective sedentary behavior. A significant overlap in the genetic variants that influence subjective and objectively measured sedentary behavior would bode well for large scale epidemiological studies and studies aiming to find genomic regions that contribute to the heritable trait variation: subjective and objectively measured sedentary behavior safely mixed in to increase the large samples sized needed for these endeavors.

Finally, there is ongoing debate on whether the risks of sedentary behavior may not simply converge with the risks of not meeting physical activity recommendations (referred to as physical inactivity), as both sedentary behavior and physical inactivity contribute to the burden of chronic diseases.^8^ The current literature seems to argue against this idea because there is only a modest relationship between MVPA and sedentary behavior.^16^, ^17^, ^18^ However, lower levels of MVPA, like higher levels of sedentary behavior, have shown to increase the risk of cardiovascular disease, type 2 diabetes, and certain types of cancer.^19^, ^20^ MVPA has repeatedly been shown to be a heritable trait in adults, although again only a few studies have used objective measurements.^12^, ^21^, ^22^ Whether the genetic risk factors influencing MVPA largely overlap with the genetic factors influencing sedentary time remains untested.

The classical twin model, in which the heritability of a trait is estimated, can be extended to a bivariate model in order to disentangle the genetic and environmental contribution to the relationship between two traits—whether they are two different measurement strategies (subjective, objective) or two different lifestyle behaviors (sitting, MVPA). In this study, the first aim was to estimate the heritability of sedentary behavior assessed by self‐report questionnaire and by a hip‐worn accelerometer in a sample of Dutch twins. The second aim was to determine the relationship between subjectively and objectively measured sedentary behavior, as well as the contribution of genetic and environmental factors to this relationship. Third, we estimated the extent to which the set of genetic factors influencing objectively measured MVPA overlaps with the set of genetic factors influencing objectively measured sedentary behavior. A potential difference between sedentary time in occupational and non‐occupational settings may exist. The former can be assumed to be less under the volitional control of the participant than the latter, which can give rise to a much larger person‐specific (work‐related) environmental effect. As an ancillary set of analyses, we therefore repeated the analyses separately for the objective assessments of occupational and non‐occupational sedentary time.

## 2 MATERIALS AND METHODS

### 2.1 Participants

This study included participants enrolled in longitudinal survey studies of the Netherlands Twin Register (NTR). The NTR was setup more than 30 years ago at the Vrije Universiteit in Amsterdam. The NTR aims, among others, to examine the underlying causes of individual differences in personality, growth, development, disease, and risk factors for disease. For the majority of the multiples and their families (parents, siblings, and children), longitudinal data are available. At this moment, more than 200 000 individuals are enrolled in the NTR. The sample described here comprises of NTR participants for which 7‐day accelerometer data were available (N = 800, aged 16‐71, 73.9% female). Accelerometer data were collected in three NTR samples; 49 monozygotic twin pairs aged 16‐26, selected based on their exercise status in their adolescence (data collected in 2013); 15 female monozygotic twin pairs participated in a study on obesity and food reward regulation^23^ (data collected in 2014‐2015); and 672 individuals recruited for a study on the determinants of sedentary behavior (data collected in 2016‐2017). All accelerometer data were collected using the same protocol (albeit embedded within different study designs) with the same instructions (see below) for every participants. 70% of this sample (N = 561) completed the short version of the International Physical Activity Questionnaire‐Short Form (IPAQ‐SF)^24^ following the 7‐day wear period of the accelerometer. In order to be eligible for the study, participants had to be physically capable of performing a normal range of daily activities (no injuries or physical handicap). The protocols for all three of the studies were approved by the VU University Medical Centre ethics committee and performed in accordance with the Declaration of Helsinki. All participants provided written informed consent.

### 2.2 Measures

#### 2.2.1 Accelerometer

Subjects wore an Actigraph accelerometer (Actigraph GT3X+, Actigraph LLC) attached to an elastic belt on the right hip during waking hours for 7 consecutive days, except during water‐based activities. Recorded data were analyzed using Actilife software (version 6.10.4). The Actigraph provides activity counts based on movement over a single‐axis. Non‐wear time was excluded and defined as 60 consecutive minutes with zero counts, with allowance of 2 minutes with counts between 0 and 100 within that time range. Wear time was considered acceptable when there was a minimum of 4 days of at least 10 hours of wear time per day. Existing cut‐points were used to define sedentary (<100 counts/min), moderate (2020‐5998 counts/min), and vigorous (>5999 counts/min)‐intensity physical activity based on the counts provided by movement over a single‐axis.^25^ The latter two were combined into one MVPA category. Sedentary time and MVPA were recalculated as percentage of total wear time. This percentage was used for subsequent analyses. Participants were asked to indicate (using a paper‐pencil form) for each day whether it was a workday (eg a day on which work is performed as distinguished from a day off, excluding study time for students) or not and if so, at what time they started and ended their workday. These time‐points were used to classify total sedentary time and MVPA into occupational time and non‐occupational time. When unreadable, unclear or no start or end times were provided, these days were excluded from the occupational/non‐occupational analyses.

#### 2.2.2 Self‐report

Following the 7‐day wear period of the accelerometer, participants completed the short version of the IPAQ to assess sitting on a typical weekday and over the weekend. Average sitting time per day was calculated as (weekday sitting minutes*5 + weekend day sitting minutes*2)/7. Total sitting time was truncated at 960 minutes/day (16 hours) following the IPAQ cleaning‐manual (www.ipaq.ki.se). Throughout this paper, self‐reported sitting time is used to quantify self‐reported sedentary time. In addition, the IPAQ‐SF assesses total physical activity over the previous week, by prompting walking, other moderate‐intensity physical activity and vigorous‐intensity physical activity. According to the IPAQ‐SF scoring manual, cases in which the sum of walking, moderate, and vigorous was greater than 960 min per week were excluded as outliers. Also, each intensity domain (walking, moderate, vigorous) exceeding 180 min per day was truncated at a duration of 180 min per day. The minutes spent on walking, moderate activity, and vigorous activity were summed to calculate the total time spent on MVPA in minutes per day.

### 2.3 Statistical analyses

Twin studies are based on the comparison of similarity of MZ twins and DZ twins. When the similarity (quantified by the correlation) between MZ twins is higher than for DZ twins, this constitutes evidence for genetic effects on the trait of interest. The relative importance of these “latent” genetic and environmental factors can be derived by structural equation modeling from observed covariances of both twin types.^26^ Genetic structural equation modeling was done in OpenMx^27^ under R (R Development Core Team, 2011) with the raw‐data ML procedure for estimation of parameters. Since (non‐twin) siblings share, like DZ twins, on average 50% of their segregating genes, parameter estimates were constrained to be equal for DZ twins and siblings. Main effects of sex and age on mean levels were considered in the model.

To estimate the heritability of sedentary behavior (first aim of the study), a so‐called saturated model was fitted, in which both the MZ and the DZ/sibling correlations of *objective* sedentary behavior (measured with the accelerometer) were estimated. Thereafter, total phenotypic variance of objective sedentary behavior was decomposed into sources of additive genetic variance (A), dominant genetic variance (variance due to non‐additive genetic effects, D) or shared environmental variance (C), and unique, or person‐specific, environmental variance (E) to test which sources of variance significantly contribute to the phenotype and estimate their most likely value. Since C and D effects cannot be estimated simultaneously in the classical twin model, the ratio of the MZ correlations to the DZ correlations was used to determine which model (ACE or ADE) was most appropriate. The significance of the estimated variance components was tested by comparing the model including the specific component to a model in which the component is constraint to be equal to zero. These nested submodels were compared by hierarchic *χ* ^2^ tests. The *χ* ^2^ statistic is computed by subtracting log‐likelihood (–2LL) for a reduced model from the −2LL for the full model (*χ* ^2^ = −2LL_full model_ – −2LL_reduced model_). This *χ* ^2^ statistic is distributed with degrees of freedom (*df*) equal to the difference in the number of parameters estimated in the two models (Δ*df* = *df*_full model_ – *df*_reduced model_). If the difference test is significant, the constraints on the reduced model cause a significant deterioration of the model fit.

**FIGURE 1:**
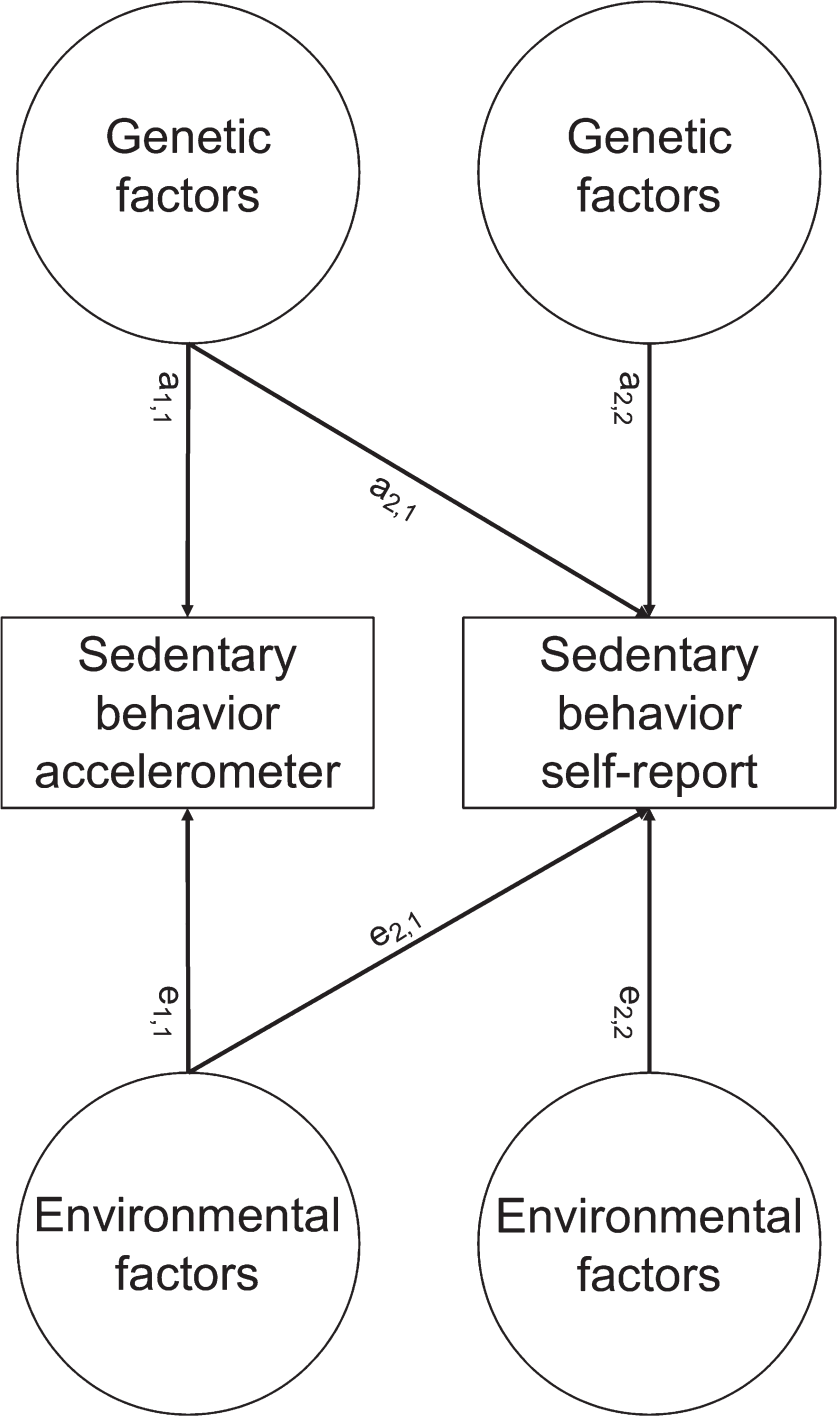
Bivariate (co)variance decomposition. A, latent additive factor; E, latent environmental factor

The second aim of the study was to determine the relationship between subjectively and objectively measured sedentary behavior. Therefore, the univariate model was extended to a bivariate model, an analysis of two variables, to determine the relationship between them, by including both objective sedentary behavior (measured with the accelerometer) and self‐reported sitting time. The phenotypic correlation between objective sedentary time and self‐reported sitting time was estimated, as well as cross‐twin/cross‐trait correlations. Subsequently, covariances of these traits were decomposed into sources of A, C or D, and E to test which sources of variance significantly contribute to the phenotypic covariance. Figure 1 shows this bivariate variance decomposition, which reveals insight into the etiology of covariances between these traits. For clarification, in Figure 1, only the latent genetic (A) and environmental (E) factors are shown (C and D are omitted). The pathway coefficients between A and E and the observed variables can be used to calculate how much of the covariance can be explained by genetics and environmental factors. For example, the total variance in phenotype 1 is calculated as *a_1,1_* ^2^ (the genetic variance, also known as the heritability) + *e_1,1_* ^2^ (variance that is explained by environmental factors). Total covariance between phenotype 1 and 2 is computed as a_1,1_*a_2,1_ (which is the genetic covariance) + e_1,1_*e_2,1_ (which represents the covariance that is explained by environmental factors). By decomposing the variance and covariance in sources of genetic and environmental factors, it is possible to estimate multivariate heritability. In addition, genetic (*r*_A_) and environmental (*r*_E_) correlations were calculated to determine how much of the genetic influence and environmental influences on two variables is common to both.

This bivariate approach was repeated including the variables objective sedentary time and objective MVPA to estimate the extent to which the genetic factors influencing objectively measured sedentary behavior correlate with the genetic factors influencing objectively measured MVPA (third aim of the study).

As information was available on whether the test‐days were working days or not, the analyses were repeated for sedentary time and MVPA in occupational as well as non‐occupational settings.

## 3 RESULTS

### 3.1 Descriptives

The final dataset consisted of 800 subjects aged 16‐71 years: 178 complete MZ twin pairs (of which 11 pairs participated with 1 sibling and 1 pair with 2 siblings) and 70 complete DZ pairs (of which 6 pairs participated with 1 sibling). In addition, 13 non‐twin sibling pairs participated (without a twin) and 259 singletons (without a participating co‐twin or sibling). For 623 participants, information was available on whether the assessed days were working days or not. Table 1 shows the descriptives of the total sample as well as the means and standard deviations of the total wear time, time spent in sedentary time, and physical activity (descriptives by twin status are listed in Table S1). Total sedentary time as measured by the accelerometers was slightly higher than self‐reported sitting time. Time spent on MVPA was overestimated by the participants, the number of minutes per day spent on MVPA measured by the accelerometer was much lower. There were significant main effects of sex on mean levels of all accelerometer‐derived variables (*P* < .01), except for non‐occupational sedentary time and non‐occupational MVPA. Total sedentary time and total MVPA was higher in males than in females. No significant age effects were detected. Further modeling allowed different mean estimates for males and females. Table S2 provides the univariate model fitting results for total and (non)‐occupational objective sedentary behavior and MVPA, as well as subjective sitting time. The following paragraphs discuss the correlations and heritability estimates from the bivariate analyses.

**TABLE 1:** General descriptives of the total sample

	Males (26.1%)		Females (73.9%)	
	Mean	SD	Mean	SD
Age	32.64	8.35	32.63	8.83
Anthropometrics				
Height (cm)	183.58	7.27	170.70	6.40
Weight (kg)	80.73	11.45	66.87	11.19
BMI (kg∙m^−1^)	23.86	3.05	22.88	3.52
Educational attainment	**%**		**%**	
Secondary schooling	4%		7%	
Lower vocational schooling	1%		1%	
Intermediate/higher vocational schooling	43%		39%	
University	27%		22%	
Unknown or not finished	25%		31%	

	Mean	SD	%^b^	Mean	SD	%^b^
*Accelerometer* ^a^						
Total—Wear time	889	69		873	62	
Total—Sedentary time	588	86	66%	554	73	63%
Total—MVPA	36	21	4%	29	18	3%
Occupational—Wear time	504	94		457	110	
Occupational—Sedentary time	355	103	70%	306	108	67%
Occupational—MVPA	18	17	4%	10	9	2%
Non‐occupational—Wear time	429	89		458	109	
Non‐occupational—Sedentary time	274	66	64%	284	71	62%
Non‐occupational—MVPA	17	13	4%	17	15	4%
*Self‐report* ^a^						
Total—Sitting time	576	163		528	153	
Total—MVPA	92	83		90	80	

^a^ Minutes per day.

^b^ Percentage of wear time.

### 3.2 The heritability of objective sedentary behavior & self‐reported sitting time

The upper two panels of Table 2 show the MZ and DZ/sibling twin correlations of objective sedentary time and self‐reported sedentary time (diagonals). The MZ correlations for sedentary time (0.58 for objective and 0.28 for self‐reported sedentary time) were higher than the DZ correlations (0.17 and 0.10, respectively), suggesting the presence of genetic factors influencing these phenotypes. Moreover, the MZ correlations were more than twice as high as the DZ correlations, suggesting dominant genetic effects (D). However, these dominant genetic effects (D) were not significant (*Χ* ^2^ = 0.38, *P* = .943) and were removed from the model, in favor of the more parsimonious AE model. The diagonals of the lower two panels of Table 2 show the percentage of the variance in objective sedentary time and self‐reported sitting time that can be explained by genetic influences (A) and environmental influences (E). For objective sedentary time, the heritability was 56% (95% CI: 44%, 65%), whereas for self‐reported sitting time it was only 26%. (95% CI: 0%, 51%). The remaining variance in the two sedentary time measures could be explained by unique environmental influences which includes measurement error.

**TABLE 2:** Upper two panels: MZ and DZ correlations and cross‐twin/cross‐trait correlations and their 95% confidence intervals in brackets for sedentary time measured with the accelerometer (objective sedentary time) and sitting time measured with the IPAQ (self‐reported sitting time). Lower two panels: standardized genetic and environmental (co)variances

	Objective sedentary time	Self‐reported sitting time
	MZ correlations	
Objective sedentary time	0.58 (0.47, 0.67)	
Self‐reported sitting time	0.39 (0.28, 0.48)	0.28 (−0.04, 0.53)
	DZ/sibling correlations	
Objective sedentary time	0.17 (−0.05, 0.37)	
Self‐reported sitting time	0.17 (0.00, 0.33)	0.10 (−0.27, 0.43)
	Genetic influences (A)	
Objective sedentary time	56% (44%, 65%)	
Self‐reported sitting time	45% (6%, 78%)	26% (0%, 51%)
	Environmental influences (E)	
Objective sedentary time	44% (35%, 56%)	
Self‐reported sitting time	55% (22%, 94%)	74% (48%, 100%)

The (phenotypic) correlation between objective sedentary time and self‐reported sitting time was only modest (*r* = 0.33, 95% CI: 0.23, 0.42). The off‐diagonals in the upper two panels of Table 2 show the cross‐twin/cross‐trait correlations. The MZ cross‐twin/cross‐trait correlation of 0.39 was higher than the DZ/sibling cross‐twin/cross‐trait correlation (0.17). Forty‐five percent of the phenotypic correlation is accounted for by genetic factors. Furthermore, a significant genetic correlation between objective sedentary time and self‐reported sitting time was found, *r*_G_ = 0.49 (95% CI: 0.09, 1.00). This genetic correlation indicates that part of the underlying genes that influence objectively assessed sedentary time also influences self‐reported sitting time. However, there are also other genetic variants that are exclusively associated with either objective sedentary or subjective sitting time. The environmental correlation between objective and self‐reported sedentary time was also significant, *r*_E_ = 0.39 (95% CI: 0.17, 0.58).

### 3.3 Objective sedentary time & objective MVPA

The (phenotypic) correlation between sedentary time and MVPA was modest but significant, *r* = −0.27 (95% CI: −0.34, −0.20). This indicates that higher levels of sedentary time were associated with lower levels of MVPA. Like objective sedentary time, objective MVPA showed to be a heritable trait with a heritability estimate of 46% (95% CI: 35%, 57%, Table S3). The correlation between objective sedentary time and objective MVPA was driven by genetic variants, explaining 59% of the overlap between these heritable traits. The genetic correlation indicates that these variants have opposite effects (*r*_G_ = −0.31, 95% CI: −0.48, −0.14), thus decreased MVPA and increased sedentary time or vice versa. The nonshared environmental factors influencing both traits are also in the opposite direction (*r*_E_ = −0.24 (95% CI: −0.37, −0.10).

Of note, *self‐reported* MVPA (measured using the IPAQ) showed a significantly lower heritability estimate of 14% (95% CI: 0%, 36%, Table S3) compared with the estimate for accelerometer‐derived objective MVPA (46%). The phenotypic correlation between self‐reported MVPA and objective MVPA was low, *r* = 0.11 (95% CI: 0.01, 0,20).

### 3.4 Occupational versus non‐occupational settings

MZ and DZ/sibling correlations (upper panel Table 3) showed similar results for occupational sedentary time (0.51 and 0.18) as for total sedentary time (0.58 and 0.17, Table 2). This resulted in a heritability estimate of 45% (95% CI: 30%, 58%) for occupational sedentary time (lower panel Table 3) which, in contrast to our expectation, is quite similar and not *lower* than the heritability estimate of total sedentary time (56%). Lower heritability was instead found for non‐occupational sedentary time (28%, 95% CI: 11%, 44%, Table 4) compared with both total and occupational sedentary time.

**TABLE 3:** Upper two panels: MZ and DZ correlations and cross‐twin/cross‐trait correlations and their 95% confidence intervals in brackets for occupational sedentary time and occupational MVPA. Lower two panels: standardized genetic and environmental (co)variances

	Objective occupational sedentary time	Objective occupational MVPA
	MZ correlations	
Objective occupational sedentary time	0.51 (0.37, 0.63)	
Objective occupational MVPA	−0.21 (−0.30, −0.13)	0.24 (0.04, 0.41)
	DZ/sibling correlations	
Objective occupational sedentary time	0.18 (−0.03, 0.38)	
Objective occupational MVPA	−0.15 (−0.27, −0.03)	0.13 (−0.11 0.33)
	Genetic influences (A)	
Objective occupational sedentary time	45% (30%, 58%)	
Objective occupational MVPA	13% (0%, 55%)	23% (0%, 42%)
	Environmental influences (E)	
Objective occupational sedentary time	55% (42%, 70%)	
Objective occupational MVPA	87% (45%, 100%)	77% (58%, 100%)

**TABLE 4:** Upper two panels: MZ and DZ correlations and cross‐twin/cross‐trait correlations and their 95% confidence intervals in brackets for non‐occupational sedentary time and non‐occupational MVPA (as assessed by the accelerometer and diary). Lower two panels: standardized genetic and environmental (co)variances

	Objective non‐occupational sedentary time	Objective non‐occupational MVPA
	MZ correlations	
Objective non‐occupational sedentary time	0.37 (0.21, 0.51)	
Objective non‐occupational MVPA	−0.36 (−0.44, −0.28)	0.47 (0.32, 0.59)
	DZ correlations	
Objective non‐occupational sedentary time	0.09 (−0.13, 0.29)	
Objective non‐occupational MVPA	−0.18 (−0.30, −0.08)	0.02 (−0.19, 0.23)
	Genetic influences (A)	
Objective non‐occupational sedentary time	28% (11%, 44%)	
Objective non‐occupational MVPA	27% (0%, 54%)	37% (20%, 51%)
	Environmental influences (E)	
Sedentary non‐occupational sedentary time	72% (56%, 89%)	
Objective non‐occupational MVPA	73% (45%, 100%)	63% (49%, 80%)

The phenotypic correlation between objective occupational sedentary time and occupational MVPA was modest (*r* = −0.29; 95% CI: −0.26, −0.21) and largely due to environmental factors (87%, lower panel of Table 3). The phenotypic correlation between objective non‐occupational sedentary time and objective non‐occupational MVPA was higher (*r* = −0.42; 95% CI: −0.48, −0.35) but also largely due to environmental factors (73%, lower panel of Table 4).

## 4 DISCUSSION

The main aim of the current study was to extend the scarce literature on the heritability of objective sedentary behavior. A relatively large sample of adult male and female twins and their siblings was equipped with an Actigraph for 7 consecutive days and reported on their sitting time and time spent on MPVA activities. We showed that more than half of the individual differences in objectively measured sedentary time could be attributed to genetic differences (56%). This estimate is comparable to the previously reported estimate of 47% for sedentary time measured objectively using a combined heart rate and movement sensor in an older, mostly female population.^15^

The heritability estimate for self‐reported sitting time in Dutch adults was much lower (26%) than that for the objective measure, but in good keeping with the heritability of 35% for total self‐reported sitting time in a cohort of older (aged 53‐67) Finnish adults.^11^ The lower heritability of self‐reported sitting time might in part be explained by recall bias, social desirability bias, or other measurement bias, which in the model are part of the unique environmental component (E), thereby inflating the influence of E. The unstandardized environmental variance in self‐reported sitting time was indeed higher compared with the environmental variance component of objective sedentary time. Another explanation for the lower heritability of our self‐report measure compared with objectively measured sedentary time is that the IPAQ‐questionnaire assesses only sitting time, while objectively measured sedentary time will also include time lying down (daytime napping) and standing still.

A few attempts have been made to identify the genetic variants underlying the heritability of self‐reported sedentary behaviors. In the Québec Family Study, a variant of the melanocortin‐4 receptor (*MC4R*) gene was found to be associated with a combined measure of self‐reported sedentary time and physical inactivity^28^ and in the Framingham Heart study an association of the fat mass and obesity‐associated (FTO) gene with sitting time was reported.^29^ These candidate gene studies are now understood to have been underpowered and confirmation through meta‐analysis of genome‐wide association (GWA) studies in very large samples from multiple cohorts are direly needed.^21^ A GWA study using accelerometer data of ~100 000 participants from the UK Biobank cohort reported 4 loci for sedentary time (rs26579 near MEF2C‐AS2, rs25981 near EFNA5, rs1858242 near LOC105377146; and rs34858520 near CALN1).^30^

If self‐reported sitting time and objectively measured sedentary time could be safely mixed in meta‐analyses it would become easier to accrue the sample sizes needed to identify the many genetic variants that may play a role in this complex and likely polygenetic behavioral trait. This requires a significant overlap in the genetic variants that influence self‐reported and objectively measured sedentary time. Our results are mildly encouraging for such future endeavors. Previous studies had shown mixed results when comparing accelerometer‐based sedentary time to survey derived sedentary time ranging from poor to reasonably strong agreement.^31^, ^32^ At the phenotypic level, we find a significant but modest correlation of *r* = 0.33 which fits this pattern of results. However, a bivariate genetic decomposition of the phenotypic correlation showed that the genetic variants that are associated with self‐reported sitting are also associated with objectively measured sedentary behavior. Although the phenotypic correlation is modest only, the genetic correlation (*r*_G_ = 0.49) might support meta‐analysis across both types of measures in genome‐wide gene‐finding studies; at least a part of the genetic variants relevant to sedentary behavior will be associated with both measures.

Recently, the Sedentary Behavior Research Network (SBRN; a network connecting sedentary behavior researchers and health professionals from around the world) updated its definitions on, among others, sedentary behavior and physical inactivity and thereby supported that an insufficient physical activity level is not the same as sedentary behavior.^1^ This idea is supported by the modest inverse correlations detected between objective sedentary time and MVPA in this study, matching those observed in earlier studies.^16^, ^17^, ^18^ This confirms that sedentary time is not simply the inverse of MVPA. Interestingly, the observed association between occupational sedentary time and occupational MVPA is −0.29, whereas the association between non‐occupational sedentary time and non‐occupational MVPA increases to −0.42, suggesting that outside work time, the association between MVPA and sedentary time is stronger. As a large portion of this is leisure time, it might be that when given the free choice, people who do more physical activity, also sit less.

When dividing total accelerometer wear time into occupational and non‐occupational time, the heritability estimate of occupational sedentary time (45%) is higher than the heritability estimate for non‐occupational sedentary time (28%). This was unexpected as sitting time at work was considered to be more under external environmental rather than under the internal control of behavioral disposition. However, sitting time is known to be strongly associated with type of work with white‐collars generally accumulating higher levels of sedentary behavior than blue collars.^33^ The type of work will be strongly dependent on educational attainment which has shown to be a heritable trait.^34^ Possibly, the genetic factors that are associated with educational attainment or other traits that co‐determine the employment setting might contribute to the variation in occupational sedentary time.

The heritability of objectively measured MVPA was 46%. This heritability is comparable to the previously reported estimates of 47% for objective MVPA, based on a combined heart rate and movement sensor^15^ and estimates of 55% and of 47% for MPA and VPA measured with an accelerometer.^35^ Self‐reported MVPA from the IPAQ showed a low heritability estimate (14%) and this echoes reports of low heritability of self‐reported MVPA in other adult samples.^36^, ^37^ Not surprisingly, we detected only a small correlation of *r* = 0.11 between objective and self‐reported MVPA. The latter may suffer from a larger measurement error because it includes a broad range of commuting, work, and household activities for which both intensity and duration may be hard to recall. Lee et al (2011) conducted a systematic review of the validity of the IPAQ‐SF and displayed that correlations between VPA/MPA/walking and objective standards showed great variability, ranging from −0.18 to 0.76.^38^ When self‐report is limited to voluntary leisure time activities of moderate‐to‐vigorous intensity, recall seems to be better. Regular exercise behavior, which is arguably easier to recall than all daily MVPA due to the mostly organized nature of exercise, has shown to be heritable in many adult twin and family samples with estimates higher than 40%.^39^, ^40^

Some limitations of this study must be noted. The twin sample used in the current study was relatively highly educated and 75% of the sample were female, which limits generalizability to the general population. This could have reduced the variance in our sample, and therefore have influenced our estimates. In addition, because of the small number of male participants we were unable to stratify our analyses by gender. Finally, as some people nowadays have flexible working hours (working part time or from home), it might be difficult to indicate their workday start and finishing times in the diary we used. Estimates of the genetic contribution to the (co)variance in occupational and non‐occupational sedentary time and MVPA might increase when a stricter distinction between working hours and non‐working hours is achieved as measurement error is reduced.

## 5 PERSPECTIVE

The majority of the variance in objectively measured sedentary time in a large sample of Dutch twins and their siblings could be explained by genetic factors. As opposed to general beliefs regarding the heritability of health behaviors, high heritability estimates do not signal that interventions are wasted efforts. Interventions on behavioral traits which are proven to be hereditary can have a large mean effect. Biological influences on the trait might explain the variation or individual differences in effect. The key is to identify the individuals who benefit the most from the intervention and exploit these biological influences on sedentary behavior in personalized or stratified interventions. Heritability studies serve to remind us that there is a biological component to individual differences in behavior. Sedentary time is not an exception. Interventions ignoring this underlying biology may prove less effective than those that build on furthering our understanding of the pathways from the genomic level to health behaviors.

## Supporting information

Table S1

Table S2

Table S3
