# Supplementary material for: Heritability of objectively assessed and self‐reported sedentary behavior
Source: Scand J Med Sci Sports. 2020 Apr 6;30(7):1237–47. doi: 10.1111/sms.13658 (PMC7318597; doi:10.1111/sms.13658)
Supplement: Supplementary file 1 — Table S1 [file SMS-30-1237-s001.docx]

Supplementary Table 1. General descriptives of the total sample per twin status

|  |  | MZ | | | | DZ | | | | Singletons† | | | |
| --- | --- | --- | --- | --- | --- | --- | --- | --- | --- | --- | --- | --- | --- |
|  |  | Males | | Females | | Males | | Females | | Males | | Females | |
|  |  | *Mean* | *SD* | *Mean* | *SD* | *Mean* | *SD* | *Mean* | *SD* | *Mean* | *SD* | *Mean* | *SD* |
| *Age* |  | 32.13 | 8.61 | 32.45 | 9.24 | 33.17 | 7.32 | 32.97 | 7.35 | 37.60 | 6.18 | 34.83 | 6.57 |
|  |  |  |  |  |  |  |  |  |  |  |  |  |  |
| *Anthropometrics* | Height (cm) | 183.50 | 7.03 | 170.20 | 6.10 | 183.54 | 6.14 | 172.36 | 6.91 | 184.40 | 11.39 | 167.67 | 29.43 |
|  | Weight (kg) | 79.89 | 13.52 | 66.18 | 11.18 | 80.47 | 11.30 | 68.78 | 13.13 | 84.32 | 7.60 | 66.58 | 15.55 |
|  | BMI (kg∙m^-1^) | 23.75 | 2.96 | 22.83 | 3.43 | 23.82 | 2.88 | 23.10 | 3.92 | 25.05 | 3.98 | 23.03 | 3.44 |
|  |  |  |  |  |  |  |  |  |  |  |  |  |  |
|  |  | *%* | | *%* | | *%* | | *%* | | *%* | | *%* | |
| *Educational* | Secondary schooling | 4 | | 8 | | 2 | | 0 | | 7 | | 14 | |
| *attainment* | Lower vocational schooling | 1 | | 1 | | 0 | | 1 | | 0 | | 0 | |
|  | Intermediate/higher vocational schooling | 36 | | 39 | | 48 | | 33 | | 53 | | 42 | |
|  | University | 25 | | 19 | | 29 | | 32 | | 20 | | 35 | |
|  | Unknown or not finished | 33 | | 34 | | 21 | | 35 | | 20 | | 19 | |
|  |  |  |  |  |  |  |  |  |  |  |  |  |  |
|  |  | *Mean* | *SD* | *Mean* | *SD* | *Mean* | *SD* | *Mean* | *SD* | *Mean* | *SD* | *Mean* | *SD* |
| *Accelerometer‡* | Total ⎯ Wear time | 892 | 68 | 870 | 63 | 879 | 73 | 876 | 58 | 897 | 61 | 889 | 58 |
|  | Total ⎯ Sedentary time | 587 | 91 | 553 | 74 | 583 | 73 | 558 | 71 | 608 | 67 | 552 | 67 |
|  | Total ⎯ MVPA | 37 | 22 | 30 | 18 | 30 | 18 | 26 | 17 | 36 | 17 | 31 | 22 |
|  | Occupational ⎯ Wear time | 507 | 93 | 456 | 110 | 493 | 105 | 448 | 108 | 517 | 51 | 482 | 115 |
|  | Occupational ⎯ Sedentary time | 354 | 102 | 304 | 108 | 352 | 113 | 305 | 106 | 380 | 60 | 326 | 108 |
|  | Occupational ⎯ MVPA | 19 | 17 | 10 | 9 | 15 | 18 | 11 | 10 | 21 | 17 | 10 | 8 |
|  | Non-occupational ⎯ Wear time | 431 | 87 | 457 | 107 | 427 | 99 | 463 | 111 | 424 | 63 | 454 | 123 |
|  | Non-occupational ⎯Sedentary time | 275 | 68 | 284 | 72 | 271 | 64 | 290 | 65 | 276 | 49 | 274 | 80 |
|  | Non-occupational ⎯MVPA | 18 | 14 | 18 | 16 | 14 | 10 | 15 | 13 | 15 | 7 | 17 | 14 |
|  |  |  |  |  |  |  |  |  |  |  |  |  |  |
| *Self report‡* | Total ⎯ Sitting time | 570 | 159 | 526 | 153 | 558 | 173 | 539 | 166 | 643 | 134 | 515 | 115 |
|  | Total ⎯ MVPA | 69 | 77 | 71 | 74 | 75 | 67 | 75 | 68 | 69 | 89 | 71 | 56 |

*Note.* †participants that are not part of a multiple (i.e. born as singleton)*;* ‡minutes per day.
