# Supplementary material for: Heritability of objectively assessed and self‐reported sedentary behavior
Source: Scand J Med Sci Sports. 2020 Apr 6;30(7):1237–47. doi: 10.1111/sms.13658 (PMC7318597; doi:10.1111/sms.13658)
Supplement: Supplementary file 2 — Table S2 [file SMS-30-1237-s002.docx]

Supplementary Table 2. Univariate model fitting results.

| Model | Variance components | | | |  | Model fit | | | |
| --- | --- | --- | --- | --- | --- | --- | --- | --- | --- |
|  | A | C | D | E | *h^2^* | -2LL | *df* | *p* | AIC |
| Objective sedentary time |  |  |  |  |  |  |  |  |  |
| ADE | 0.31 (0.00, 0.64) |  | 0.25 (0.00, 0.65) | 0.44 (0.35, 0.55) | 56% | 5481.01 | 777 |  | 3927.01 |
| **AE** | **0.56 (0.44, 0.65)** |  |  | **0.44 (0.35, 0.58)** | **56%** | **5481.28** | **778** | **.601** | **3925.28** |
| DE |  |  | 0.56 (0.45, 0.65) | 0.44 (0.35, 0.55) | 56% | 5481.40 | 778 | .534 | 3925.40 |
| E |  |  |  | 1.00 | 0% | 5538.28 | 779 | < .001 | 3980.28 |
| Objective occupational sedentary time | |  |  |  |  |  |  |  |  |
| ADE | 0.16 (0.00, 0.58) |  | 0.32 (0.00, 0.60) | 0.52 (0.40, 0.67) | 48% | 5139.19 | 606 |  | 3927.19 |
| AE | 0.47 (0.32, 0.59) |  |  | 0.53 (0.41, 0.68) | 47% | 5139.37 | 607 | .505 | 3925.37 |
| **DE** |  |  | **0.49 (0.34, 0.60)** | **0.51 (0.40, 0.66)** | **49%** | **5139.30** | **607** | **.744** | **3925.30** |
| E |  |  |  | 1 | 0% | 5170.94 | 608 | < .001 | 3954.94 |
| Objective non-occupational sedentary time | |  |  |  |  |  |  |  |  |
| ADE | 0.00 (0.00, 0.00) |  | 0.28 (0.00, 0.43) | 0.72 (0.57, 0.91) | 28% | 4305.61 | 606 |  | 3093.61 |
| AE | 0.25 (0.07, 0.41) |  |  | 0.75 (0.59, 0.93) | 25% | 4306.36 | 607 | .387 | 3092.36 |
| **DE** |  |  | **0.28 (0.09, 0.43)** | **0.72 (0.57, 0.91)** | **28%** | **4305.61** | **607** | **.999** | **3091.61** |
| E |  |  |  | 1 | 0% | 4313.88 | 608 | < .001 | 3097.88 |
| Objective MVPA |  |  |  |  |  |  |  |  |  |
| ADE | 0.27 (0.00, 0.56) |  | 0.21 (0.00, 0.57) | 0.53 (0.43, 0.64) | 48% | 6943.45 | 777 |  | 5389.45 |
| **AE** | **0.47 (0.35, 0.57)** |  |  | **0.53 (0.43, 0.65)** | **47%** | **6943.63** | **778** | **.666** | **5387.63** |
| DE |  |  | 0.48 (0.36, 0.57) | 0.52 (0.43, 0.64) | 48% | 6943.73 | 778 | .595 | 5387.73 |
| E |  |  | 1 |  | 0% | 6991.65 | 779 | < .001 | 5433.65 |
| Objective occupational MVPA | | |  |  |  |  |  |  |  |
| ADE | 0.00 (0.00, 0.41) |  | 0.26 (0.00, 0.45) | 0.74 (0.55, 0.99) | 26% | 2893.20 | 606 |  | 1681.20 |
| AE | 0.23 (0.00, 0.42) |  |  | 0.77 (0.58, 0.99) | 23% | 2893.48 | 607 | .596 | 1679.48 |
| **DE** |  |  | **0.26 (0.01, 0.45)** | **0.74 (0.55, 0.99)** | **26%** | **2893.20** | **607** | **1** | **1679.20** |
| E |  |  |  | 1 | 0% | 2897.39 | 608 | .040 | 1681.39 |
| Objective non-occupational MVPA | |  |  |  |  |  |  |  |  |
| ADE | 0.00 (0.00, 0.00) |  | 0.40 (0.00, 0.53) | 0.60 (0.47, 0.77) | 40% | 3097.75 | 606 |  | 1885.75 |
| **AE** | **0.38 (0.21, 0.51)** |  |  | **0.62 (0.49, 0.79)** | **38%** | **3097.22** | **607** | **.225** | **1885.22** |
| DE |  |  | 0.40 (0.23, 0.53) | 0.60 (0.47, 0.77) | 40% | 3097.75 | 607 | 1 | 1883.75 |
| E |  |  |  | 1 | 0% | 3117.03 | 608 | < .001 | 1901.03 |
| Self-reported sitting time |  |  |  |  |  |  |  |  |  |
| ACE | 0.14 (0.00, 0.47) | 0.02 (0.00, 0.36) |  | 0.84 (0.53, 1) | 14% | 2728.33 | 321 |  | 2086.33 |
| AE | 0.16 (0.00, 0.47) |  |  | 0.84 (0.53, 1) | 16% | 2728.33 | 322 | .959 | 2084.33 |
| CE |  | 0.11 (0.00, 0.36) |  | 0.89 (0.64, 1) | 0% | 2728.39 | 322 | .805 | 2084.39 |
| **E** |  |  |  | **1** | **0%** | **2729.03** | **323** | **.425** | **2083.03** |
| Self-reported MVPA |  |  |  |  |  |  |  |  |  |
| ADE | 0.00 (0.00, 0.00) |  | 0.22 (0.00, 0.43) | 0.78 (0.57, 1) | 22% | 5051.82 | 427 |  | 4197.82 |
| AE | 0.19 (0.00, 0.41) |  |  | 0.81 (0.59, 0.99) | 19% | 5052.50 | 428 | 0.410 | 4196.50 |
| DE |  |  | 0.22 (0.00, 0.43) | 0.78 (0.57, 1) | 22% | 5051.82 | 428 | 1 | 4195.82 |
| **E** |  |  |  | **1** | **0%** | **5054.90** | **429** | **0.080** | **4196.90** |

Note. A=additive genetic variance, C=shared environmental variance, D=dominant genetic variance, E=unique, or person-specific, environmental variance, *h^h^*=heritability, -2LL= log-likelihood, *df*=degrees of freedom, AIC=Akaike information criterion.
