# Supplementary material for: Heritability of objectively assessed and self‐reported sedentary behavior
Source: Scand J Med Sci Sports. 2020 Apr 6;30(7):1237–47. doi: 10.1111/sms.13658 (PMC7318597; doi:10.1111/sms.13658)
Supplement: Supplementary file 3 — Table S3 [file SMS-30-1237-s003.docx]

Supplementary Table 3. Upper two panels: MZ and DZ correlations and cross-twin/cross-trait correlations for MVPA measured with the accelerometer (objective MVPA) and MVPA behavior measured with the IPAQ (self-reported MVPA). Lower two panels: standardized genetic and environmental (co)variances.

|  | Objective MVPA | Self-reported MVPA | |
| --- | --- | --- | --- |
|  | MZ correlations | | |
| Objective MVPA | 0.49 (0.37 0.60) | |  |
| Self-reported MVPA | 0.10 (-0.02, 0.23) | | 0.21 (0.02, 0.50) |
|  | DZ/sibling correlations | | |
| Objective MVPA | 0.16 (-0.07, 0.37) | |  |
| Self-reported MVPA | 0.15 (0.02, 0.27) | | -0.16 (-0.52, 0.13) |
|  | Genetic influences (A) | | |
| Objective MVPA | 46% (35%, 57%) | |  |
| Self-reported MVPA | 0% | | 14% (0%, 36%) |
|  | Environmental influences (E) | | |
| Objective MVPA | 54% (44%, 65%) | |  |
| Self-reported MVPA | 100% | | 86% (64%, 100%) |
